# Evaluating shear wave elastography for differentiating lipomas from low to intermediate grade liposarcomas: is it reproducible and reliable?

**DOI:** 10.1007/s00256-025-04960-z

**Published:** 2025-06-02

**Authors:** Michelle Wei Xin Ooi, Michelle Wilson, Jonathan Nicholls, Bill Pass, Harun Gupta, Elizabeth Hensor, Philip Robinson

**Affiliations:** 1 Musculoskeletal Centre X-Ray Department, Leeds Teaching Hospitals Trust, Chapel Allerton Hospital, Chapeltown Road, Leeds, LS7 4SA UK; 2 Leeds Institute of Rheumatic and Musculoskeletal Medicine, University of Leeds, Leeds, UK; 3 NIHR Leeds Biomedical Research Centre, Leeds, UK; 4 Airedale NHS Foundation Trust, West Yorkshire, UK

**Keywords:** Shear wave elastography, Ultrasound, Magnetic resonance imaging, Lipoma, Liposarcoma, Well-differentiated liposarcoma

## Abstract

**Objective:**

To evaluate if imaging findings and shear wave elastography (SWE) can differentiate between lipoma and low-to-intermediate grade liposarcoma and to assess for SWE reproducibility across different manufacturer machines.

**Materials and methods:**

Consecutive patients referred for lipomatous soft tissue mass biopsy prospectively underwent B-mode ultrasound (US), SWE on two machines (LOGIQ-E9 and SSI-Aixplorer), and MRI. Three musculoskeletal radiologists independently scored images and gave an assessment of malignancy on a four-point scale. Inter-reader agreement was evaluated with kappa statistic (US/US + MRI) and ICC (SSI-Aixplorer velocity/stiffness; cross-machine comparisons). Diagnostic utility of shear wave velocity (SWV) was evaluated using area under receiver operating characteristic (AUROC) curve against pathology.

**Results:**

Among 269 patients (mean age 58.8 ± 15.7 years, 34.6% (93/269) female), 22% (59/269) of lesions were malignant. Inter-reader agreement on SSI-Aixplorer was good for velocity and stiffness with ICC 0.87 (95% CI 0.75–0.94) and 0.93 (95% CI 0.87–0.97) respectively. Agreement between machines was moderate for velocity and stiffness with ICC 0.62 (95% CI 0.53–0.69) and 0.66 (95% CI 0.58–0.73) respectively. SWV poorly predicted malignancy (AUROC 0.57, 95% CI 0.49–0.65), and failed to differentiate benign from malignant lipomatous tumours at previously defined 2.02 m/s threshold, misclassifying 9.5% (4/42) as benign. SWV failed to further stratify radiologist grouped benign/probably benign lesions on US (AUROC 0.59, 95% CI 0.49–0.68). Using MRI to further stratify lesions classified as benign/probably benign on US with SWV ≤ 2.02 m/s had a sensitivity of 61.8% and specificity of 80.2%.

**Conclusion:**

SWE did not enhance diagnostic accuracy in differentiating lipoma from low-to-intermediate grade liposarcoma and lacked reproducibility across different machines.

**Supplementary Information:**

The online version contains supplementary material available at 10.1007/s00256-025-04960-z.

## Introduction

Lipomas and low-grade (grade 1) or well-differentiated liposarcomas (WDLPS) often appear similar on conventional ultrasound and MRI, posing a diagnostic challenge. Lipomas are benign adipose tumours without cellular atypia and are the most common soft tissue tumour (nearly 50% of all soft tissue tumours) [1, 2]. Liposarcomas make up about 15–20% of soft tissue sarcomas with the most common subtype being WDLPS [3–6]. WDLPS are often painless, slow growing, locally aggressive masses without metastatic risk but may dedifferentiate into a more aggressive and malignant subtype. Whilst several imaging observations such as large size > 10 cm, presence of thick septa, and nodularity have been described as suggesting WDLPS, these findings are non-specific on their own with a significant number of lipomas and WDLPS appearing similar [7–9]. Diagnostic accuracy in differentiating between benign lipoma and WDLPS is limited for ultrasound (49–64%) [10] and MRI (69–73%) [11, 12], often necessitating biopsy which is invasive and carries risk of bleeding, infection, and sampling error. Histopathology can be non-specific, requiring fluorescence in situ hybridisation (FISH) to detect MDM2 gene amplification, which distinguishes WDLPS (MDM2-positive) from lipoma [13]. Benign lipoma can be managed conservatively with surgical removal typically performed for cosmetic or symptomatic reasons. WDLPS requires surgical removal usually by marginal excision for cosmesis and function preservation which can be curative but there are reports of high local recurrence rate (ranging between 8.9 and 52%) suggesting wide excision and adjuvant radiotherapy to minimise local recurrence risk [14–17].

Shear-wave elastography (SWE) is a non-invasive ultrasound technique that obtains a quantitative measure of the elasticity or stiffness of the imaged tissues by measuring the propagation velocity of the shear waves produced [18, 19]. SWE has been shown to be useful in differentiating between benign and malignant lesions in several organs such as breast, thyroid, and prostate [20–25]. Studies in soft tissue sarcoma remain inconclusive with small sample size and/or heterogenous studies including multiple sarcoma subtypes [26–30].

This study aims to examine whether SWE is able to help differentiate lipoma and WDLPS and therefore reduce the need for invasive biopsy. It also aims to compare SWE measurements of lipomatous soft tissue lesions between different vendor machines.

## Materials and methods

### Patient population

**Fig. 1:**
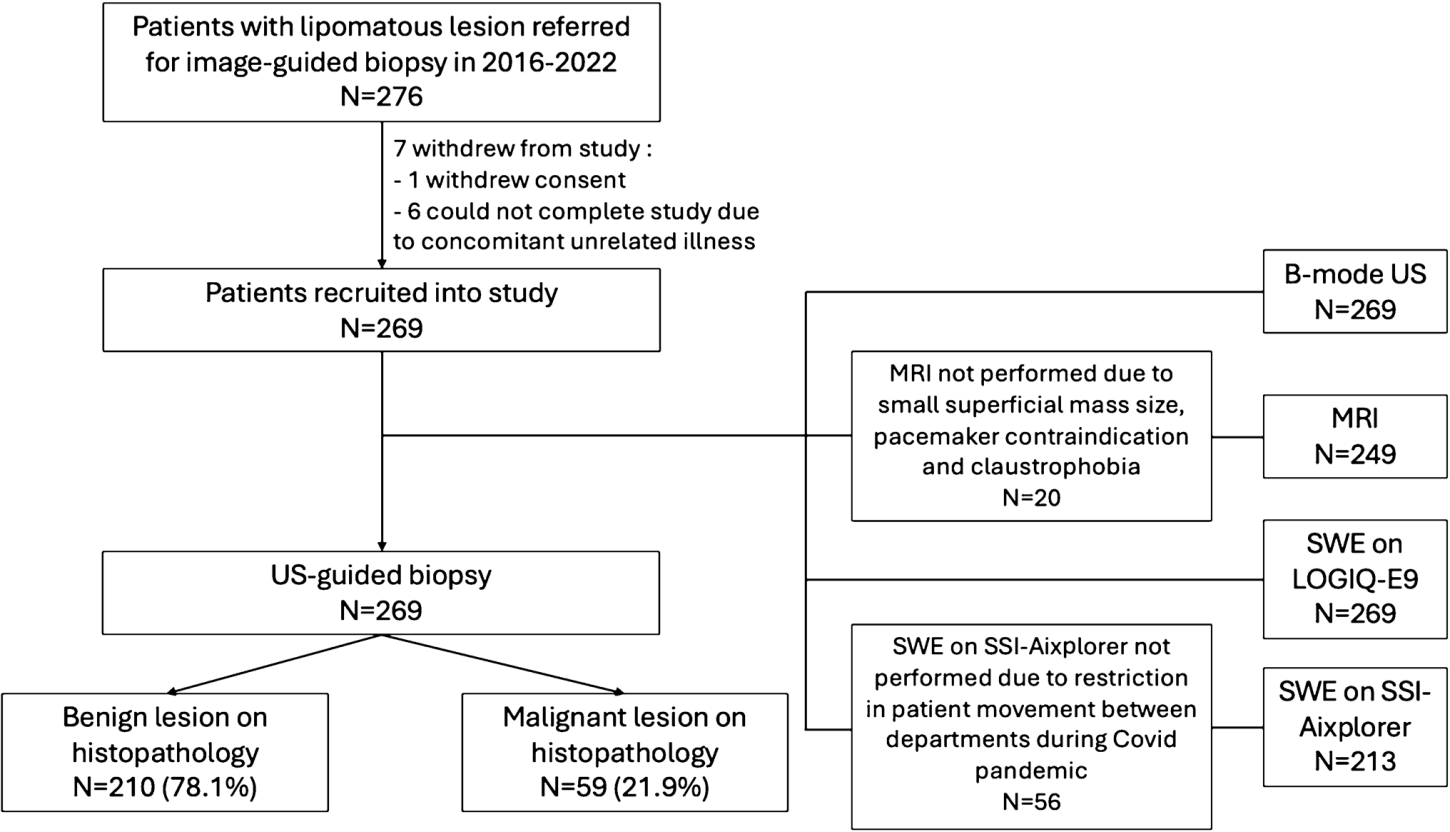
Flowchart of participants in the study. SWE shear wave elastography

The study was approved by the institutional ethics review board, and all procedures performed were in accordance with the ethical standards of the institution and with the 1964 Helsinki declaration and its later amendments. Written informed consent was obtained from all patients. The study prospectively recruited 276 consecutive patients with a lipomatous lesion referred from the regional soft tissue sarcoma service for image-guided biopsy between May 2016 and August 2022 to determine if the lesion is a lipoma versus WDLPS. The inclusion criteria consisted of lipomatous lesions without overtly aggressive features (e.g. large infiltrative masses with minimal/no macroscopic fat, or evidence of osseous/muscular invasion). Whilst this selection inherently introduces bias by excluding lesions with non-fatty characteristics, it creates a clinically relevant cohort for study. Notably, the absence of distinguishing features between benign and malignant lesions was expected, as the inclusion criteria inherently precluded such differentiation. Seven patients withdrew from the study (1 patient withdrew consent and 6 could not complete the study protocol due to unrelated concomitant illnesses), leaving 269 patients recruited into the study (Fig. 1). All patients recruited into the study had image-guided biopsy. All biopsies were performed percutaneously using a minimum 16-gauge core needle to obtain at least three cores. Histologic evaluation (histopathology and MDM2 amplification genetic testing) of the biopsy result or surgical specimen (where available) was used as the reference standard, with pathologic diagnosis confirmed after analysis by one of two specialist soft-tissue sarcoma pathologists.

### B-mode ultrasound

All patients underwent B-mode ultrasound examination, performed by one of three musculoskeletal radiologists (BP, HG, and PR with 12, 15, and 27 years of experience respectively) using a 6–15 MHz linear transducer (LOGIQ-E9, General Electric Healthcare, Milwaukee, WI, USA). Visual inspection was used to categorise the lesions into one of four categories: benign, probably benign, probably malignant, or malignant based on qualitative assessment using previously published criteria [30, 31]. The anonymized images were also reviewed by the two other radiologists who performed a visual assessment of malignancy using the same four-point categorical scale (“US-only”). Ultrasound features recorded were heterogenicity (yes/no), necrosis (yes/no), echotexture (hypoechoic, hyperechoic, mixed), Doppler flow (absent, linear, disorganised), nodule (yes/no), nodule content (fatty and/or calcified, or non-fatty), nodule size (< 1 cm or > 1 cm), septi (yes/no), and septi thickness (≤ 2 mm or > 2 mm). Radiologists were blinded to any MRI study results if available.

### MRI

MRI was performed using a 1.5-T system (Magnetom Avanto, Siemens Healthcare, Erlangen, Germany) in 249 patients prior to biopsy. Twenty patients did not have an MRI performed for clinical reasons including small superficial mass size (< 2 cm), pacemaker contraindication, and/or claustrophobia. T1-weighted and T2 fat-saturated images were obtained in two planes, without intravenous contrast medium. All three radiologists provided a second assessment of malignancy on the same four-point categorical scale after reviewing both the anonymised US and MRI images (“US + MRI”). MRI features recorded were lesion fat content (100%, 75–99% or < 75%), heterogeneity (present, linear oedema, or non-nodular oedema), nodule (yes/no), nodule content (fatty and/or calcification, or non-fatty), nodule size (< 1 cm or > 1 cm), nodule oedema (yes/no), septi (yes/no), septi thickness (≤ 2 mm or > 2 mm), and septi oedema (yes/no). This was performed at least 3 weeks after the US-only scoring.

For assessments based on US only and US + MRI, analyses used the majority score of the three independently obtained scores without discussion or further analysis by radiologists (see statistical analysis below).

### Ultrasound shear wave elastography (SWE)

All patients recruited were consented for two-dimensional SWE on two different vendor machines before biopsy: LOGIQ-E9 (General Electric Healthcare, Milwaukee, WI, USA) and SSI-Aixplorer (SuperSonic Imagine, Aix-en-Provence, France). All 269 patients had SWE performed on the LOGIQ-E9 machine. Due to the COVID pandemic, 56/269 patients could not undergo SWE on the SSI-Aixplorer machine as patient movement between the different hospital department ultrasound rooms was restricted, and therefore 213/269 patients had SWE on both machines.

**Fig. 2:**
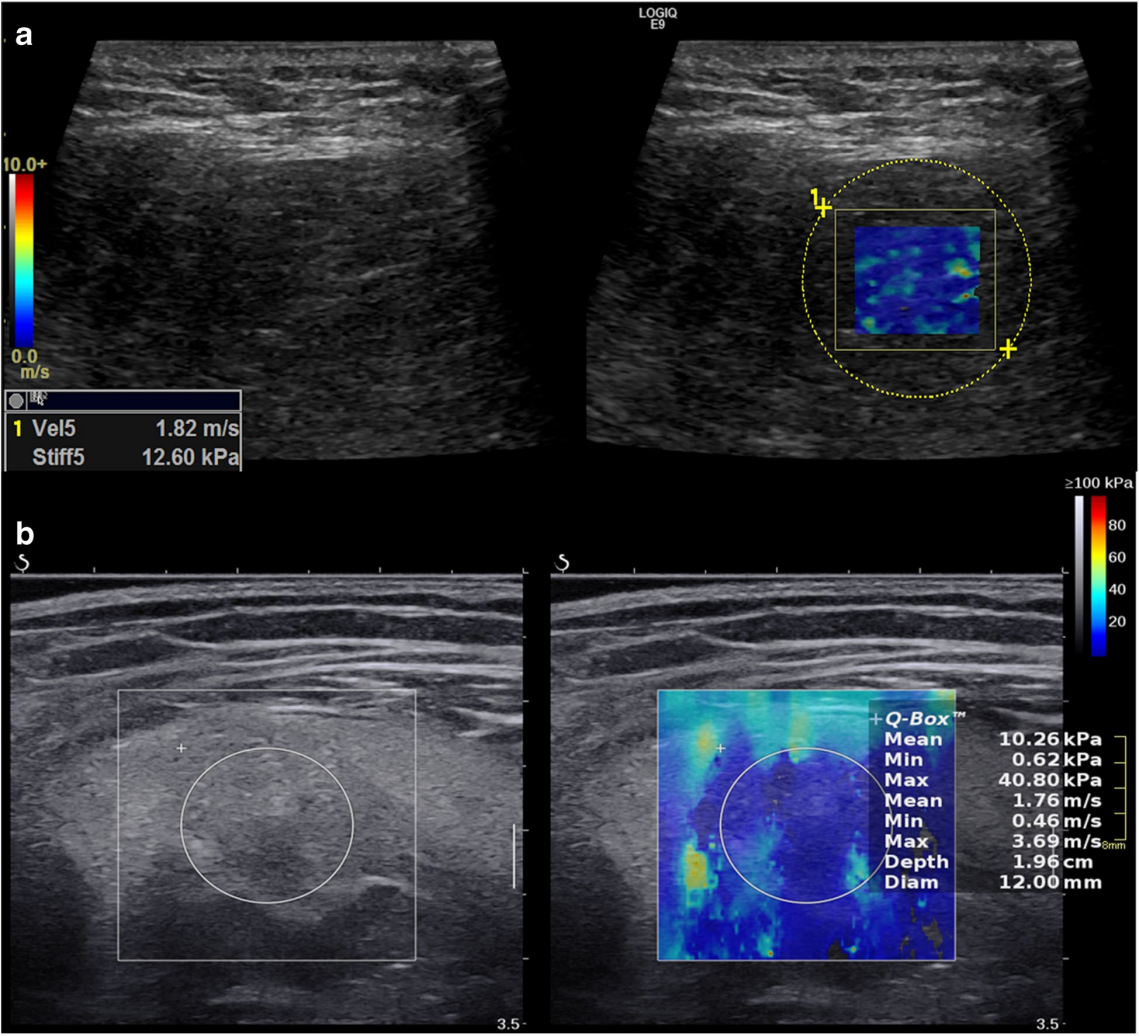
Shear wave elastography in **a** 47-year**-**old male patient with MDM2 negative benign lipoma on GE LOGIQ-E9 machine and **b** 67**-**year**-**old male patient with well differentiated liposarcoma (WDLPS) on SSI-Aixplorer machine

The ultrasound systems both report SWE readings in shear wave velocity (SWV) [metres/seconds (m/s)] and stiffness, Young’s modulus [kilopascals (kPa)]. Young’s modulus is calculated by the system using the following equation: *E* = 3ρVs^2^ where *E* is Young’s modulus, *ρ* is tissue density (assumed to be 1 g/cm^3^), and Vs is the velocity of shear waves. Both units are proportional to elasticity and can be used as a surrogate for tissue stiffness. All SWE examinations were obtained using a previous validated protocol [27, 30]. Patients were placed in relaxed positions depending on the location of the lesion. The positions were adjusted to ensure no active (contraction) or passive (stretching) effects directly influenced the elasticity results. The SWE rectangular elasticity box was fixed at a size of 1.5 cm × 2 cm and a circular region of interest (ROI) was utilised to cover and calculate elasticity within the box in the most solid and vascularised area (Fig. 2). Selected SWE maps were free from random inconsistent artefactual colour patterns. Five repeated measurements were obtained in the transverse plane of the lesion, and an average of the five measurements was taken. SWE readings on the LOGIQ-E9 and SSI-Aixplorer machines were acquired by one of three musculoskeletal radiologists (authors BP, HG, and PR with > 5 years’ experience with SWE each) [30].

In 28 cases, SWE readings on the SSI-Aixplorer were also acquired by author PR and a board-qualified sonographer (> 6 years in musculoskeletal SWE) to evaluate inter-reader reproducibility. Inter-reader reproducibility on the LOGIQ-E9 machine had been evaluated previously in the same institution [30].

### Statistical analysis

All analyses were performed using R version 4.4.2 in R Studio [32, 33]. Sample size determination was conducted for various statistical analyses including inter-rater reliability Cohen’s kappa, Bland and Altman plot, interclass correlation coefficient (ICC), area under receiving operator curve (AUROC), and sensitivity and specificity. The largest minimum sample size needed was 264 with details of each calculation criteria and method [34–37] presented in Supplementary Table 1. Multirater reliability was assessed using unweighted kappa statistic with 95% confidence intervals (CI) via R function KappaM [38]. To assess radiologists’ agreement over US-only and US + MRI classification of lesions, proportions of agreement with corresponding 95% CIs were calculated using function agreement [39]. Kappa may underestimate agreement due to its assumptions about rater independence; thus, percentage agreement was also calculated as it has been suggested to be a more reliable measure of interrater reliability in well-trained subjects with minimal guessing [40]. To compare log-transformed measurements taken on LOGIQ-E9 with those taken on SSI-Aixplorer, limits of agreement were extracted and anti-logged with Bland–Altman plots constructed using functions bland.altman and ggplot [41, 42]. To assess measurement reliability, ICC was calculated using function icc [43]. To assess the predictive performance of US classification (benign/probably benign vs probably malignant/malignant), sensitivity, specificity, and positive/negative predictive values were calculated using function epi.tests [44]. To evaluate the ability SWV to correctly classify lesions, AUROC with corresponding 95% CIs (2000 bootstraps) was calculated using function roc [36], applying previously defined thresholds [30] to current data for validation.

## Results

A total of 269 patients were recruited into the study (Fig. 1); the mean age was 58.8 years (standard deviation (SD) 15.7) and 93/269 (34.6%) were female (Table 1). Histopathology confirmed that 59/269 lesions (21.9%) were malignant. Lipoma (138/269 (51.3%)) and low grade (grade 1) liposarcoma (47/269 (17.5%)) were the most common benign and malignant lesions found respectively, and 12/269 (4.5%) lesions were intermediate-grade (grade 2) liposarcoma (Supplementary Table 2).

**Table 1:** Participant demographic characteristics and B-mode ultrasound features split by biopsy-confirmed benign and malignant lipomatous mass

		Total (*n* = 269)	Benign (*n* = 210)	Malignant (*n* = 59)
Age	Mean (SD)	58.8 (15.7)	56.5 (15.6)	67.3 (13.1)
	Median (range)	60 (18, 89)	57 (18, 89)	69 (25, 88)
Sex	Female	93 (34.6%)	72 (34.3%)	21 (35.6%)
Ultrasound features				
Heterogeneity	Present	186 (69.1%)	141 (67.1%)	45 (76.3%)
Necrosis	Present	15 (5.6%)	7 (3.3%)	8 (13.6%)
Echotexture	Hypoechoic	39 (14.5%)	29 (13.8%)	10 (16.9%)
	Hyperechoic	128 (47.6%)	109 (51.9%)	19 (32.2%)
	Mixed	102 (37.9%)	72 (34.3%)	30 (50.8%)
Doppler flow	Absent	167 (62.1%)	137 (65.2%)	30 (50.8%)
	Linear	87 (32.3%)	64 (30.5%)	23 (39.0%)
	Disorganised	15 (5.6%)	9 (4.3%)	6 (10.2%)
Nodule	Present	37 (13.8%)	28 (13.3%)	9 (15.3%)
	Nodule (fat/calcified)	16 (43.2%)	12 (42.9%)	4 (44.4%)
	Nodule (non-fat)	24 (64.9%)	17 (60.7%)	7 (77.8%)
	Nodule < 1 cm	15 (40.5%)	12 (42.9%)	3 (33.3%)
	Nodule > 1 cm	22 (59.5%)	16 (57.1%)	6 (66.7%)
Septi	Present	266 (98.9%)	207 (98.6%)	59 (100.0%)
	Septi thin (≤ 2 mm)	258 (97.0%)	201 (97.1%)	57 (96.6%)
	Septi thick (> 2 mm)	10 (3.8%)	7 (3.4%)	3 (5.1%)
Malignancy (based on consensus of three readers)	Benign	85 (31.6%)	76 (36.2%)	9 (15.3%)
	Probably benign	130 (48.3%)	99 (47.1%)	31 (52.5%)
	Probably malignant	48 (17.8%)	34 (16.2%)	14 (23.7%)
	Malignant	6 (2.2%)	1 (0.5%)	5 (8.5%)
Size CC (cm)	Mean (SD)	11.0 (7.3)	9.5 (6.5)	16.7 (7.3)
	Median (range)	8.8 (1.7, 70.3)	8.2 (1.7, 70.3)	15.1 (3.1, 37.4)
Size AP (cm)	Mean (SD)	5.7 (3.6)	5.2 (3.4)	7.5 (3.4)
	Median (range)	4.8 (1.2, 20.0)	4.1 (1.2, 18.4)	7.1 (2.1, 20.0)
Size transverse (cm)	Mean (SD)	7.2 (3.9)	6.8 (3.6)	8.7 (4.3)
	Median (range)	6.1 (1.0, 25.0)	6.0 (1.0, 25.0)	7.8 (2.1, 19.7)
Elliptical volume (mm^3^)	Mean (SD)	390.8 (698.3)	269.2 (443.6)	823.6 (1140.2)
	Median (range)	150.8 (5.1, 6505.7)	104.4 (5.1, 3063.1)	405.5 (8.2, 6505.7)
Position	Deep	157 (58.4%)	101 (48.1%)	56 (94.9%)
	Deep and subcutaneous	1 (0.4%)	1 (0.5%)	0 (0.0%)
	Subcutaneous	111 (41.3%)	108 (51.4%)	3 (5.1%)
	Deep (intra-muscular)	67 (42.4%)	39 (38.2%)	28 (50.0%)
	Deep (inter-muscular)	96 (60.8%)	63 (61.8%)	33 (58.9%)

Abbreviations: *SD* standard deviation, *size CC* craniocaudal, *size AP* anteroposterior

### US and MRI reader agreement for lesion classification

When considering grouped categories of malignancy (benign/probably benign vs probably malignant/malignant), the kappa statistic (*k*) for agreement between readers of the US results was 0.81 (95% CI (0.74, 0.89)), suggesting strong agreement [40]. The equivalent figure for the US + MRI results was 0.57 (95% CI (0.50, 0.64)), suggesting moderate agreement [45]. Overall percentage agreement for imaging findings was 94.3% (95% CI (92.1, 96.1)) for US and 79.4% (95% CI (75.3, 83.2) for US + MRI (Supplementary Table 3).

### US and MRI features in benign vs malignant lesions

Little difference was observed between benign and malignant lipomatous lesions in the majority of US and MRI features evaluated such as heterogeneity, presence of doppler flow, non-fatty nodule, nodule larger than 1 cm, and thick septi (> 2 mm) (Tables 1 and 2). Presence of necrosis on ultrasound was more common in malignant lesions (8/59 (13.6%)) compared to benign lipomas (7/210 (3.3%)). Malignant lesions were more likely to have mixed echotexture (30/59 (50.8%)) compared to benign lipomas (72/210 (34.3%)) whilst benign lipomas were more likely to appear hyperechoic (109/210 (51.9%)) compared to malignant lesions (19/59 (32.2%)).

**Table 2:** MRI features split by biopsy-confirmed benign and malignant lipomatous mass

MRI features		Total (*n* = 249)	Benign (*n* = 192)	Malignant (*n* = 57)
Fat (%)	100%	50 (20.1%)	43 (22.4%)	7 (12.3%)
	75–99%	168 (67.5%)	128 (66.7%)	40 (70.2%)
	< 75%	31 (12.4%)	21 (10.9%)	10 (17.5%)
Heterogeneity	Present	236 (94.8%)	180 (93.8%)	56 (98.2%)
	Linear oedema	103 (43.6%)	73 (40.6%)	30 (53.6%)
	Non-nodular oedema	158 (66.9%)	124 (68.9%)	34 (60.7%)
Nodule	Present	85 (34.1%)	61 (31.8%)	24 (42.1%)
	Nodule (fat/calcified)	38 (44.7%)	29 (47.5%)	9 (37.5%)
	Nodule (non-fat)	50 (58.8%)	35 (57.4%)	15 (62.5%)
	Nodule < 1 cm	19 (22.4%)	14 (23.0%)	5 (20.8%)
	Nodule > 1 cm	66 (77.6%)	47 (77.0%)	19 (79.2%)
	Nodule oedema	52 (61.2%)	36 (59.0%)	16 (66.7%)
Septi	Present	185 (74.3%)	134 (69.8%)	51 (89.5%)
	Septi thin (< 2 mm)	171 (92.4%)	124 (92.5%)	47 (92.2%)
	Septi thick (> 2 mm)	37 (20.0%)	19 (14.2%)	18 (35.3%)
	Septi oedema	86 (46.5%)	57 (42.5%)	29 (56.9%)
Malignancy (based on consensus of three readers)	Benign	35 (14.1%)	32 (16.7%)	3 (5.3%)
	Probably benign	121 (48.6%)	106 (55.2%)	15 (26.3%)
	Probably malignant	81 (32.5%)	51 (26.6%)	30 (52.6%)
	Malignant	12 (4.8%)	3 (1.6%)	9 (15.8%)

The mean elliptical volume for malignant lesions was three times larger than that of the benign group; 823.6 mm^3^ (SD = 1140.2 mm^3^) vs 269.2 mm^3^ (SD = 443.6 mm^3^) (Table 1). Within the malignant lesions, 56/59 (94.9%) and 3/59 (5.1%) were located in the deep (intramuscular or intermuscular) and subcutaneous layers respectively. Within the benign group, 101/210 (48.1%) and 108/210 (51.4%) of lesions were located in the deep (intramuscular or intermuscular) and subcutaneous layers respectively (Table 1).

### Shear wave velocity and stiffness

The mean (SD) transverse shear wave velocity (SWV) was 2.0 m/s (1.5) for benign lesions and 1.7 m/s (0.5) for malignant lesions on the LOGIQ-E9, and 1.8 m/s (0.7) for benign lesions and 1.6 m/s (0.7) for malignant lesions on the SSI-Aixplorer (Table 3). The mean (SD) transverse shear wave stiffness was 14.9 kPa (23.3) for benign lesions and 10.1 kPa (6.1) for malignant lesions on the LOGIQ-E9, and 13.0 kPa (13.0) for benign lesions and 11.7 kPa (9.8) for malignant lesions on the SSI-Aixplorer (Table 3).

**Table 3:** Ultrasound shear wave features split by biopsy-confirmed benign and malignant lipomatous mass

Shear wave continuous value (LOGIQ-E9)		Total (*n* = 269)	Benign (*n* = 210)	Malignant (*n* = 59)
Velocity (m/s)	Mean (SD)	2.0 (1.4)	2.0 (1.5)	1.7 (0.5)
	Median (range)	1.6 (1.0, 17.2)	1.6 (1.1, 17.2)	1.5 (1.0, 3.3)
Stiffness (kPa)	Mean (SD)	14.0 (21.3)	14.9 (23.3)	10.1 (6.1)
	Median (range)	8.4 (2.2, 223.8)	8.4 (2.2, 223.8)	7.9 (3.7, 34.1)
Shear wave continuous value (SSI-Aixplorer)		Total (*n* = 213)	Benign* (*n* = 175)	Malignant* (*n* = 38)
Velocity (m/s)	Mean (SD)	1.8 (0.7)	1.8 (0.7)	1.6 (0.7)
	Median (range)	1.6 (0.7, 5.9)	1.6 (0.8, 5.9)	1.6 (0.7, 3.8)
Stiffness (kPa)	Mean (SD)	12.7 (12.4)	13.0 (13.0)	11.7 (9.8)
	Median (range)	9.6 (0.3, 107.2)	9.7 (0.3, 107.2)	9.3 (1.6, 48.2)
Shear wave categorised (LOGIQ-E9)		Total (*n* = 269)	Benign* (*n* = 210)	Malignant* (*n* = 59)
Velocity (m/s)	≤ 1.78	166 (61.7%)	123 (58.6%)	43 (72.9%)
	> 1.78	103 (38.3%)	87 (41.4%)	16 (27.1%)
Velocity (m/s)	≤ 2.02	205 (76.2%)	156 (74.3%)	49 (83.1%)
	> 2.02	64 (23.8%)	54 (25.7%)	10 (16.9%)

Abbreviations: *m/s* metres/seconds, *kPa* kilopascals, *SD* standard deviation

### SWE measurements between readers and across machines

ICC for velocity and stiffness on the SSI-Aixplorer machine across two readers (*n* = 28) were 0.87 (95% CI 0.75–0.94) and 0.93 (95% CI 0.87–0.97), indicating good agreement. Most readers agreed on lesions above and below previously derived thresholds (Supplementary Table 4). Bland–Altman plots (Supplementary Fig. 1) showed that 95% of velocity measurements by reader 1 would fall between 0.70 and 1.54 times those by reader 2, and 95% of stiffness measurements by reader 1 would fall between 0.48 and 2.14 times those by reader 2.

ICC for velocity and stiffness measured across the LOGIQ-E9 and SSI-Aixplorer machines were 0.62 (95% CI 0.53–0.69) and 0.66 (95% CI 0.58–0.73), indicating moderate agreement. The two machines showed disagreement over lesions above and below previously derived thresholds (Supplementary Table 5). Bland–Altman plots (Supplementary Fig. 2) indicated that 95% of velocity measurements from the SSI-Aixplorer were between 0.59 and 1.99 times those from the LOGIQ-E9, and 95% of stiffness measurements from the SSI-Aixplorer were between 0.32 and 3.11 times those from the LOGIQ-E9.

### Does SWE add information beyond lesion assessment with US alone

Table 4 presents the predictive performance of US alone, with an NPV of 81.4% (95% CI 75.5–86.4), potentially missing 18.6% of malignant lesions. SWE was evaluated for further stratification, assuming lower SWV values indicate malignancy. Table 5 shows the predictive performance of SWE using SWV thresholds of ≤ 1.78 and ≤ 2.02 m/s. At ≤ 1.78 m/s, sensitivity and specificity were 82.5% (95% CI 67.2–92.7) and 36.6% (95% CI 29.4–44.2), respectively. At ≤ 2.02 m/s, sensitivity increased to 90% (95% CI 76.3–97.2), but specificity dropped to 21.7% (95% CI 15.8–28.6). The AUROC for SWV in benign/probably benign lesions was 0.59 (95% CI 0.49–0.68), indicating no added diagnostic value.

**Table 4:** Predictive performance of the overall ultrasound classification of probably malignant/malignant vs. probably benign/benign compared to histopathology diagnosis

US	Benign* (*n* = 210)	Malignant* (*n* = 59)	TP	FP	TN	FN	Sensitivity% (95% CI)	Specificity% (95% CI)	PPV% (95% CI)	NPV% (95% CI)
Benign	76	9	59	210	0	0	100 (93.9, 100)	0 (0, 1.7)	21.9 (17.1, 27.4)	(-)
Probably benign	99	31	50	134	76	9	84.7 (73, 92.8)	36.2 (29.7, 43.1)	27.2 (20.9, 34.2)	89.4 (80.8, 95)
Probably malignant	34	14	19	35	175	40	32.2 (20.6, 45.6)	83.3 (77.6, 88.1)	35.2 (22.7, 49.4)	81.4 (75.5, 86.4)
Malignant	1	5	5	1	209	54	8.5 (2.8, 18.7)	99.5 (97.4, 100)	83.3 (35.9, 99.6)	79.5 (74.1, 84.2)

Abbreviations: *TP* true positive, *FP* false positive, *TN* true negative, *FN* false negative, *CI* confidence interval, *PPV* positive predictive value, *NPV* negative predictive value

^*^Biopsy-confirmed

**Table 5:** Predictive performance of shear wave velocity to predict benign/malignant compared to histopathology diagnosis

Lesion set/subset	SWV threshold (m/s)	TP	FP	TN	FN	Sensitivity% (95% CI)	Specificity% (95% CI)	PPV% (95% CI)	NPV% (95% CI)
All lesions	SWV ≤ 1.78	43	123	87	16	72.9 (59.7, 83.6)	41.4 (34.7, 48.4)	25.9 (19.4, 33.3)	84.5 (76, 90.9)
Benign/probably benign on US	SWV ≤ 1.78	33	111	64	7	82.5 (67.2, 92.7)	36.6 (29.4, 44.2)	22.9 (16.3, 30.7)	90.1 (80.7, 95.9)
All lesions	SWV ≤ 2.02	49	156	54	10	83.1 (71, 91.6)	25.7 (19.9, 32.2)	23.9 (18.2, 30.3)	84.4 (73.1, 92.2)
Benign/probably benign on US	SWV ≤ 2.02	36	137	38	4	90 (76.3, 97.2)	21.7 (15.8, 28.6)	20.8 (15, 27.6)	90.5 (77.4, 97.3)

Abbreviations: *SWV* shear wave velocity, *m/s* metres/seconds, *TP* true positive, *FP* false positive, *TN* true negative, *FN* false negative, *CI* confidence interval, *PPV* positive predictive value, *NPV* negative predictive value

### Can MRI further stratify us benign/probably benign lesions with SWV ≤ 2.02 m/s?

**Fig. 3:**
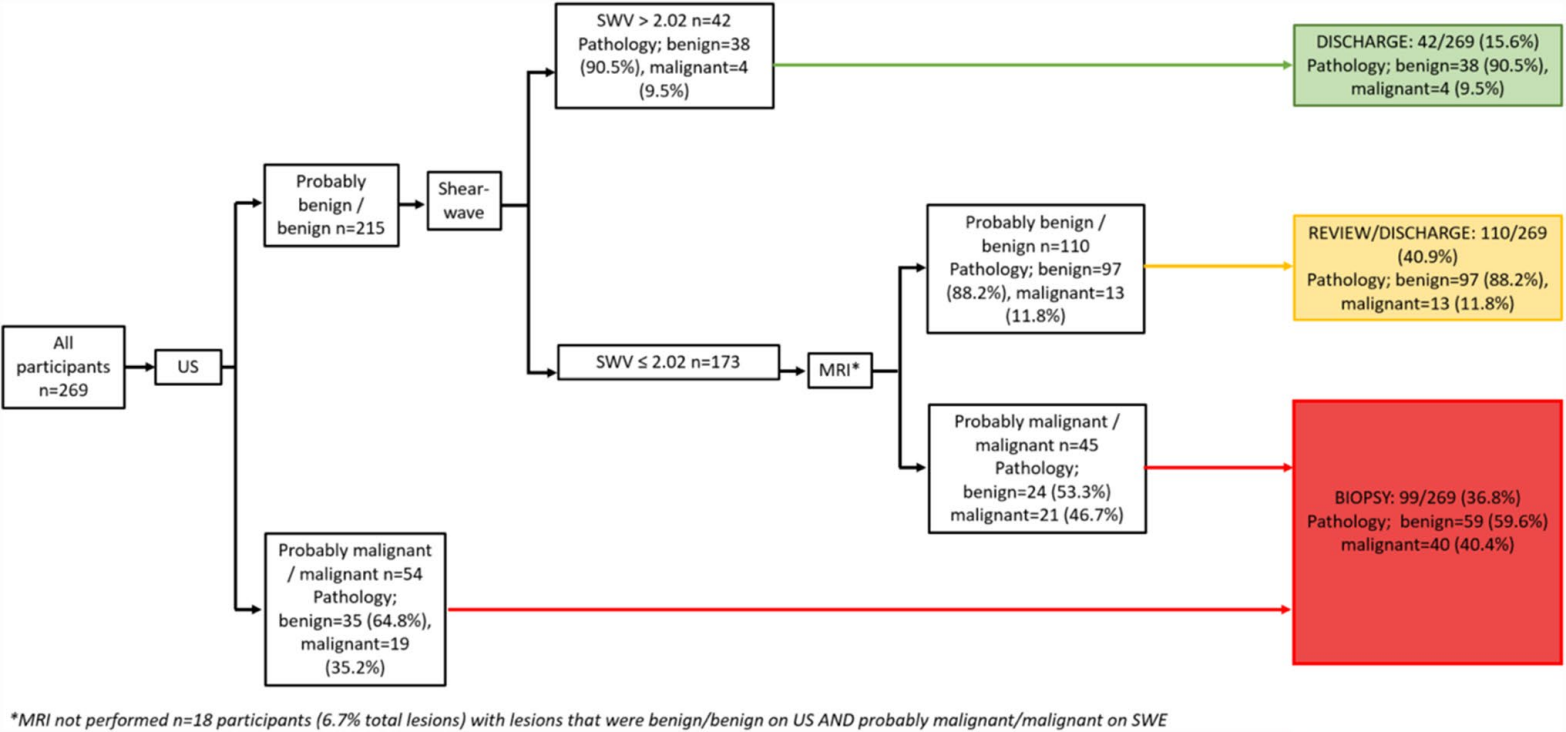
Using shear wave velocity threshold of 2.02 m/s to stratify lesions classed as probably benign/benign on US and subsequent stratification by MRI

The total number of patients with lesions classified as benign/probably benign on US with SWV ≤ 2.02 m/s was 173 (Fig. 3). The predictive values in using MRI to further stratify these lesions are presented in Supplementary Table 6 with a sensitivity and specificity rate of 61.8% and 80.2% respectively.

## Discussion

### US and MR features in benign vs malignant lesions

Whilst percentage reader agreement was 94.3% (95% CI 92.1–96.1) for US classification and 79.4% (95% CI 75.3–83.2) for MRI, conventional imaging appearances were non-specific. US and MR features classically thought to be malignant such as heterogeneity, presence of doppler flow, non-fatty nodule, nodule larger than 1 cm, and thick septi (> 2 mm) [7, 46] did not show any difference in occurrence between benign lipomas and malignant lipomatous lesions in our study. Histological grading of soft tissue sarcomas are performed according to FNCLCC grading system looking at tumour differentiation, mitotic count, and tumour necrosis [47]. With the exception of tumour necrosis, the other categories are not measurable on conventional imaging. However, we found larger lesion volume and deep location of lesion to be more reliable indicators for low-to-intermediate grade liposarcomas which is consistent with previous study findings [46, 48].

### Shear wave velocity and stiffness

Our study found slightly higher mean transverse SWV and stiffness values in benign compared to malignant lipomatous lesions. Previous SWE studies in breast, thyroid, liver, and prostate suggest that greater stiffness (higher SWV) correlates with malignancy [49, 50]. In contrast, malignant musculoskeletal soft tissue lesions tend to have lower SWVs, indicating they are softer or less stiff [27, 30, 51]. This variability may reflect the diverse histological features of musculoskeletal tumours, where calcification and fibrosis increase stiffness, whilst haemorrhage, necrosis, and cystic degeneration reduce it [52]. Our findings showed that previously defined SWE thresholds failed to distinguish benign from malignant lipomatous tumours, in line with previous studies’ conclusions that SWE was not useful in differentiating between benign and malignant soft tissue tumours [26–28]. Although Li et al. found significant SWE value differences between benign and malignant musculoskeletal soft tissue tumours, their study was limited to 21 malignant and 60 benign soft tissue lesions [29]. Biopsy and histological diagnosis remain the gold standard for differentiating between benign and malignant lipomatous soft tissue tumours [28].

### SWE measurements between readers and across machines

There was a high level of reproducibility when using the SSI-Aixplorer machine between readers with ICC 0.87 and 0.93 (95% CI 0.87–0.97) for velocity and stiffness respectively. This is similar to a previous study result in the same institution using the LOGIQ-E9 machine with ICC 0.93 (95% CI 0.89–0.95) and 0.92 (95% CI 0.88–0.94) for velocity and stiffness respectively [30]. Other studies have also validated the reproducibility of SWE in a variety of other body parts [53, 54]. However, we found SWE measurements to be considerably different when measured across the two different vendor machines which supports some of previous studies consensus [55–57] although the majority of the data measured in the literature was on phantom models. Given our inter-observer reproducibility on the same machine was good, we postulate that the variability in readings between machines could be due to differences in each company’s algorithm and technology in generating and measuring the acoustic waves [57, 58]. Therefore, caution should be exercised in adopting a quantitative cutoff value when using different vendors in clinical practice. To our knowledge, this study is the first to compare SWE readings between two different machines on patients with lipomatous soft tissue lesions.

### Does SWE add information beyond lesion assessment with US alone?

Using US alone to discharge benign or probably benign lesions yielded an NPV of 81.4% (95% CI 75.5–86.4), meaning 18.6% of malignant cases could be missed. Data on the NPV of US for differentiating benign from malignant lipomatous lesions is limited, making direct comparison difficult. The lower-than-expected NPV may reflect the study’s recruitment from a tertiary sarcoma centre and the inclusion of only lower grade lipomatous lesions without overtly aggressive imaging features, such as large infiltrative masses, minimal fat content, or bone/muscle invasion. These cases were excluded to maintain data homogeneity.

Our results showed that SWE was not able to further stratify lesions initially classified as benign or probably benign on conventional ultrasound, with an AUROC of 0.59 (95% CI (0.49, 0.68)). Several studies in breast radiology have reported usefulness of SWE in confirming that a lesion is benign and suggested that invasive biopsy can be avoided in these cases [24, 25, 59]. A previous study of soft tissue sarcomas reported that SWE could improve the diagnostic confidence that a musculoskeletal soft tissue lesion is benign although it is important to note the small proportion of lipomatous soft tissue lesions (< 30) in that study [30]. Bodard et al. separated SWE analysis between 99 non-fatty and 37 fatty tumours due to lower elasticity in fat-containing tumours, and found that SWE had 84% specificity in separating benign from malignant non-fatty soft tissue masses using a tumour-to-fat elasticity ratio value of 3.5, but results were inconclusive in fatty tumours [60].

### Can MRI further stratify US benign/probably benign lesions with SWV ≤ 2.02 m/s?

MRI stratification of lesions classified as benign/probably benign on US with SWV ≤ 2.02 m/s had a sensitivity of 61.8% and specificity of 80.2%, showing limited added value. The reported sensitivity and specificity of using ultrasound alone in diagnosing lipoma range between 52 to 95% and 86 to 99% respectively [10, 61, 62] whilst MRI detects malignant lipomatous tumours with 76 to 91% sensitivity and 37 to 79% specificity, though it is worth noting that some studies included higher grade malignant lipomatous tumours [8, 63, 64]. To the authors’ knowledge, there is no data in literature on the predictive value in using MRI to further stratify benign/probably benign lipomatous lesions on US with a low SWV value.

Study limitations include a reduced number of SSI-Aixplorer scans due to COVID restrictions, though the 269-lesion sample remains valuable for identifying any significant trends or limitations in SWE evaluation of lipomatous lesions. Selection bias is likely, as patients were recruited from specialist sarcoma centre referrals, but this is a practical approach given the rarity of liposarcomas. Whilst only non-aggressive appearing lipomatous lesions were included, a small number were later identified as intermediate grade (grade 2) liposarcomas following FNCLCC grading of whole specimen mitotic counts after resection, introducing slight heterogeneity. However, this reflects real-world challenges in distinguishing benign lipomas from low- to intermediate-grade liposarcomas on conventional imaging, underscoring the rationale for this study on the application of shear wave elastography.

## Conclusion

In this study, SWE was not able to reliably differentiate between benign and malignant lipomatous tumours or reduce biopsy rates, alone or alongside other imaging. This is the largest study on SWE in this tumour type and the only clinical study comparing its reproducibility across machines. Despite being a negative study, it provides valuable data for future research. Biopsy, histology, and MDM2 amplification remain the gold standard, whilst non-invasive methods like radiomics show promise but require further validation [63].

## Supplementary Information

Below is the link to the electronic supplementary material.

Supplementary file1 (DOCX 23 KB)

Supplementary file2 (DOCX 112 KB)

## Data Availability

Thirty study subjects (between May 2016 to March 2017) have been previously reported in Tavare AN, Alfuraih AM, Hensor EMA, Astrinakis E, Gupta H, Robinson P (2019) Shear-Wave Elastography of Benign versus Malignant Musculoskeletal Soft-Tissue Masses: Comparison with Conventional US and MRI. Radiology 290:410–417.
