# Supplementary material for: Evaluating shear wave elastography for differentiating lipomas from low to intermediate grade liposarcomas: is it reproducible and reliable?
Source: Skeletal Radiol. 2025 Jun 2;54(12):2669–80. doi: 10.1007/s00256-025-04960-z (PMC12552375; doi:10.1007/s00256-025-04960-z)
Supplement: Supplementary file 1 — Supplementary file1 (DOCX 23 KB) [file 256_2025_4960_MOESM1_ESM.docx]

# Supplementary Table 1. Sample size needed statistical calculation criteria and method

| Statistical Measure | Method & R Function | Assumptions & Parameters | Required Sample Size |
| --- | --- | --- | --- |
| Kappa | CIBinary (kappaSize package) | 35% malignancy rate, 5% significance, k = 0.7, ±0.1 precision, 3 raters | 156 |
| Bland-Altman |  |  | 100 |
| ICC | calculateIccSampleSize  (ICC.Sample.Size package) | 5% significance, 80% power, two-tailed, ICC ≥ 0.65 | 155 |
| AUROC | power.roc.test (pROC package) | 35% malignancy rate*, 5% significance, 80% power, AUROC ≥ 0.70 | 87 |
| Sensitivity / Specificity |  | 95% CI width ±5%, Sensitivity=40%, Specificity=90%, 35% malignancy rate* | 264 |

** estimated values from previous study by Tavare AN, Alfuraih AM, Hensor EMA, Astrinakis E, Gupta H, Robinson P (2019) Shear-Wave Elastography of Benign versus Malignant Musculoskeletal Soft-Tissue Masses: Comparison with Conventional US and MRI. Radiology 290:410-417*

*Abbreviations; ICC : Interclass correlation coefficient, AUROC : Area under receiver operating curve*

# Supplementary Table 2. Histopathological subgroup of lipomatous lesions

| **Histopathological subgroup** | **n = 269** |
| --- | --- |
| **Benign (n=210)** | |
| Lipoma | 138 (51.3%) |
| Lipoma with fat necrosis | 10 (3.7%) |
| Spindle cell lipoma | 32 (11.9%) |
| Hibernoma | 18 (6.7%) |
| Fibrolipoma | 6 (2.2%) |
| Angiolipoma | 2 (0.7%) |
| Fat necrosis | 2 (0.7%) |
| Pleomorphic lipoma | 2 (0.7%) |
| **Malignant (n=59)** | |
| Grade 1 liposarcoma / WDLPS | 47 (17.5%) |
| Grade 2 liposarcoma | 12 (4.5%) |

*Abbreviations; WDLPS : Well-differentiated liposarcoma*

# Supplementary Table 3. Categorical agreement between readers on US and US+MRI when classifying lesions, with benign/probably benign and probably malignant/malignant categories combined.

|  |  | **Overall % agreement (95% CI)** | **Percentage agreement (95% CI)** | **Benign / probably benign** | **Probably malignant / malignant** |
| --- | --- | --- | --- | --- | --- |
| **US** | **Benign / probably benign** | **94.3 (92.1, 96.1)** | 96.5 (94.6, 97.9) | 635 | 7 |
|  | **Probably malignant / malignant** |  | 84.6 (77.2, 90.5) | 39 | 126 |
|  |  |  |  |  |  |
| **US+MRI** | **Benign / probably benign** | **79.4 (75.3, 83.2)** | 82.9 (78.3, 87.1) | 374 | 109 |
|  | **Probably malignant / malignant** |  | 74.0 (66.7, 80.5) | 45 | 219 |

*Abbreviations; CI=confidence interval*

# Supplementary Table 4. Categorical agreement of shear wave velocity measured on the SSI-Aixplorer by two readers.

|  |  | **Reader 2** | |
| --- | --- | --- | --- |
|  |  | **≤2.02** | **>2.02** |
| **Reader 1** | **≤2.02** | 19 (16:3) | 1 (1:0) |
|  | **>2.02** | 2 (2:0) | 6 (4:2) |

*Numbers in parenthesis represent benign:malignant ratio as determined by pathology.*

# Supplementary Table 5. Categorical agreement of velocity measured on two different shear-wave machines when using the previously defined threshold.

|  |  | **SSI-Aixplorer** | |
| --- | --- | --- | --- |
|  |  | **≤2.02** | **>2.02** |
| **GE-LOGIQ** | **≤2.02** | 141 (113:28) | 24 (20:4) |
|  | **>2.02** | 16 (14:2) | 32 (28:4) |

*Numbers in parenthesis represent benign:malignant ratio as determined by pathology.*

# Supplementary Table 6. Predictive performance of MRI in lesions classified as benign/probably benign on ultrasound with shear wave velocity ≤2.02 m/sec (n=173, MRI not performed on 18 of these patients, n=155) compared to histopathology diagnosis.

|  | **TP** | **FP** | **TN** | **FN** | **Sensitivity % (95% CI)** | **Specificity % (95% CI)** | **PPV % (95% CI)** | **NPV % (95% CI)** |
| --- | --- | --- | --- | --- | --- | --- | --- | --- |
| **US benign/probably benign lesions with SWV ≤ 2.02 m/sec** | 21 | 24 | 97 | 13 | 61.8 (43.6, 77.8) | 80.2 (71.9, 86.9) | 46.7 (31.7, 62.1) | 88.2 (80.6, 93.6) |

*Abbreviations; SWV=shear wave velocity*, m/sec= meters/seconds*, TP=true positive, FP=false positive, TN=true negative, FN=false negative, CI=confidence interval, PPV=positive predictive value, NPV=negative predictive value*
