# Supplementary material for: Evaluating shear wave elastography for differentiating lipomas from low to intermediate grade liposarcomas: is it reproducible and reliable?
Source: Skeletal Radiol. 2025 Jun 2;54(12):2669–80. doi: 10.1007/s00256-025-04960-z (PMC12552375; doi:10.1007/s00256-025-04960-z)
Supplement: Supplementary file 2 — Supplementary file2 (DOCX 112 KB) [file 256_2025_4960_MOESM2_ESM.docx]

# Supplementary Fig. 1 Bland-Altman plots for level of agreement between log-transformed a) velocity and b) stiffness shear-wave measurements taken by two different readers on the SSI-Aixplorer machine


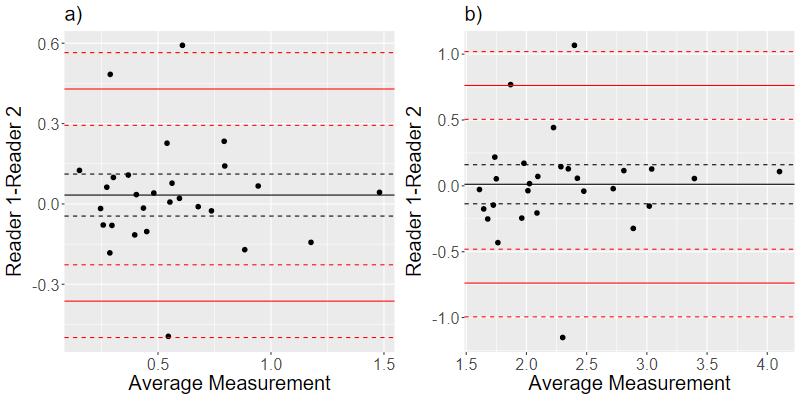


*Solid black lines represent average difference (bias), solid red lines represent limits of agreements and dashed lines represent corresponding 95% confidence intervals.*

# Supplementary Fig. 2 Bland-Altman plots for the level of agreement between log-transformed a) velocity (m/sec) and b) stiffness (kPa) shear-wave measurements taken on the SSI-Aixplorer and the GE-LOGIQ E9 machines.


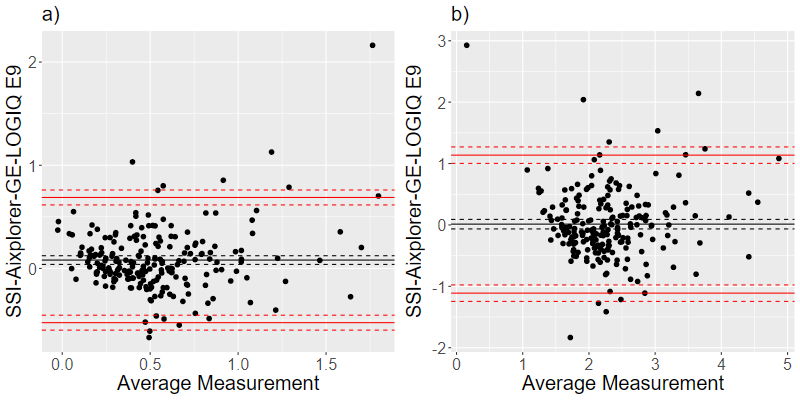


*Solid black lines represent average difference (bias), solid red lines represent limits of agreements and dashed lines represent corresponding 95% confidence intervals.*
